# Structural Architectural Features of Cyclodextrin Oligoesters Revealed by Fragmentation Mass Spectrometry Analysis

**DOI:** 10.3390/molecules23092259

**Published:** 2018-09-05

**Authors:** Cristian Peptu, Maksym Danchenko, Ľudovít Škultéty, Jaroslav Mosnáček

**Affiliations:** 1 Polymer Institute, Slovak Academy of Sciences, Dúbravská cesta 9, 84541 Bratislava, Slovakia; 2 “Petru Poni” Institute of Macromolecular Chemistry, Grigore Ghica Voda 41A, 700487 Iasi, Romania; 3 Institute of Virology, Biomedical Research Center, Slovak Academy of Sciences, Dúbravská cesta 9, 84505 Bratislava, Slovakia; virumaks@savba.sk (M.D.); viruludo@savba.sk (Ľ.Š.)

**Keywords:** cyclodextrin, oligolactide, mass spectrometry, ring-opening oligomerization, MALDI, ESI, collision-induced dissociation, laser-induced dissociation, biodegradable polymers

## Abstract

Cyclodextrins (CDs) were used in the present study for the ring-opening oligomerization (ROO) of l-lactide (LA) in order to synthesize biodegradable products with possible applications in pharmaceutical and medical fields. The practical importance of ROO reactions may reside in the possibility of synthesizing novel CD derivatives with high purity due to the dual role played by CDs, the role of the initiator through the hydroxylic groups, and the role of the catalyst by monomer inclusion in the CD cavity. The analyzed compounds were CDs modified with oligolactides obtained through ROO reactions of l-lactide in dimethylformamide. The resulting CD isomeric mixtures were investigated using classical characterization techniques such as gel permeation chromatography and nuclear magnetic resonance. Moreover, advanced mass spectrometry (MS) techniques were employed for the determination of the average number of monomer units attached to the cyclodextrin and the architecture of the derivatives (if the monomer units were attached as a single chain or as multiple chains). Thus, fragmentation studies effectuated on two different instruments (ESI Q-TOF and MALDI TOF) allowed us to correlate the size of the oligolactide chains attached to the CD with the observed fragmentation patterns.

## 1. Introduction

Cyclodextrins (CDs) are cyclic oligosaccharides with a truncated conical shape obtained by enzymatic procedures from starch. The most well-known representatives are α-, β-, and γ-CD, which have a number of 6, 7, and 8 glycoside units, respectively. Cyclodextrins have become increasingly important molecules for a wide range of chemistry-related applications, such as biomaterials, biodegradable materials, drug delivery, odor masking, or removal of organic pollutants [1,2,3].

These applications are related to capability of CD molecules to include hydrophobic guest molecules into their internal cavity by physical forces. Eventually, the CDs may require chemical modifications in order to accommodate particular needs. Therefore, the OH functions of the CDs are customizable for required organic functionalities such as methyl, 2-OH-propyl, sulfobutyl, etc., which are already available as commercial products [3]. Besides modifications with low molecular weight functionalities, CD molecules have been used as scaffolds for the synthesis of polymers with various architectures [4,5]. Polymers containing CDs are produced by “arm first” (polymer chains are grafted onto the CD molecules) or “core first” (CD plays the role of polymerization initiator) synthetic strategies using classical polymerization techniques. Also, CD molecules may be modified with polymerizable moieties and used in polymerization reactions. In a core first approach, CDs may be used as such or they may be modified in order to play the role of initiators for star polymers. There have been several studies regarding the synthesis of CD-containing polymers, such as free radical polymerization, reversible-deactivation radical polymerizations [4], as well as ring-opening polymerizations (ROP) of cyclic esters [6], oxiranes [7], and oxazolines [8].

The classical chemistry methods employed so far for polymerization of cyclic esters initiated by CDs used catalysts commonly used in ROP, such as Sn-octoate [9,10,11] or amines-based organic catalysts [12,13,14,15]. However, there is one particular polymerization reaction that uses the special inclusion properties of the CD molecules. Specifically, Harada’s pioneering work demonstrated that CD molecules may play a dual role in the polymerization of δ-valerolactone, β-butyrolactone, and ε-caprolactone [16]. First, CDs participate in the ring-opening process as an initiator, and second, monomer activation occurs by including the monomer units inside the CD cavity. These polymerizations were carried out in bulk and the reaction products consisted of complex mixtures of CD-oligoester isomers.

CD modified with oligolactide chains (CD-LA) can be synthesized by bulk [17,18] or solution polymerization [19]. CD-LA was demonstrated to be highly degradable in a water environment and may be further used for the preparation of drug inclusion complexes. The structure of CD-LA prepared in solution was found to be similar to that obtained by bulk polymerization; however, the solution procedure retains the advantage of homogenous products being obtained (no unmodified CDs, no homo-oligoesters, and fewer transesterifications reactions).

CD-oligoesters are complicated to characterize due to the structural complexity of the CD original scaffold. The structural characterization should take into account the following issues: the total number of monomer units attached to a single CD unit, the average number of monomer units per OH functionalized position (equivalent to the average length of the oligoester arms attached to the CD), and the positional isomers resulting from the place of functionalization on the CD (polymerization from the OH groups situated at the smaller or larger rim). On the other hand, the continuous search for new macromolecular compounds addressed to bio-related applications requires more sophisticated characterization tools for accurate structural analysis. Thus, mass spectrometry (MS) arises as a technique of choice to complete classical techniques such as nuclear magnetic resonance (NMR) spectroscopy. MS can resolve issues like molar mass, endgroup identification, comonomer composition, etc. [20] CD derivatives have been characterized by single-stage MALDI MS analysis or by chromatographic separation with offline [21] or online MS detection of the compounds [22,23].

The CD-oligoester derivatives have been characterized by MALDI MS [12,13,16,18] or ESI MS [15,19,24,25] for the structural assignment of the products. However, complex structure identification requires more than a single-stage MS measurement. In such situations, the structural characterization is performed by fragmentation experiments called tandem MS (MS/MS). Detection and interpretation of the fragmentation spectra ions allows reconstruction of the primary structure (connectivity) of the selected polymer architecture in the case of polyesters [26,27,28,29]. Such fragmentation studies were applied in the case of CD modified with 3-OH-butyrates [15,25] obtained by ROP of β-butyrolactone catalyzed by (−)-sparteine.

The current work envisages the mass spectrometry study, MS and MS/MS, of custom-synthesized CD-LA. Commonly, controlled fragmentations for analytical purposes are performed in mass spectrometers with an electrospray ionization source. However, the mass range in which the single charged ions can be produced usually does not exceed *m*/*z* = 2000, depending on the instrumental setup and the chemical nature of the analyzed sample. The compounds exceeding this *m*/*z* value may appear as doubly or multiply charged species in the MS spectrum. Although such multiply charged species may be also fragmented, the resulting spectra are complicated and the obtained practical information is frequently less useful than in the study of singly charged species. Thus, we intend to demonstrate that MALDI MS/MS may represent a solution in analyzing singly charged CD derivatives with higher masses. The prepared CD-LA sample was characterized by ^1^H NMR and gel permeation chromatography (GPC) in order to have a clear structural image and, then, MS techniques were employed in order to have a deeper understanding of the structural properties. Moreover, the collision-induced dissociation (CID) fragmentation patterns were in a first approach established using a high-resolution ESI Q-TOF instrument and, then, the MALDI MS/MS (laser-induced dissociation-LID) profiles were established for higher molecular weight components of the CD-LA sample.

## 2. Results and Discussion

**Scheme 1:**
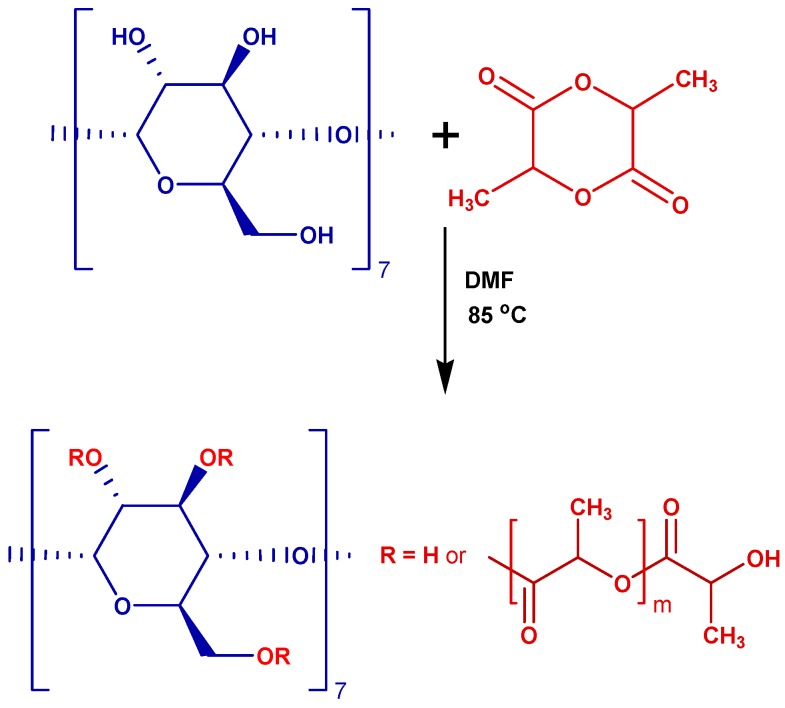
Ring opening of l-lactide (LA) in the presence of cyclodextrins (CDs).

The CD-LA derivatives taken into consideration in this study were obtained using a synthetic procedure (Scheme 1) previously proposed by Shen et al. [19]. In their study, they proposed the synthesis of CD derivatives with a low content of oligolactides, about 5 lactate **(la)** units per CD molecule. The ROP process was possibly catalyzed by DMF at 85 °C. Herein, we targeted the attachment of a higher amount, 16 lactate units per CD molecule, by a simple increase of l-lactide (LA)-to-CD feed ratio. The obtained products were characterized by NMR, GPC, MALDI MS, and ESI/MALDI MS/MS.

### 2.1. Nuclear Magnetic Resonance (NMR) and Gel Permeation Chromatography (GPC) Characterization

The ^1^H NMR analysis may give information about the structure of the compounds (chemical nature), about the site of the esterification (at the OH groups from the second, third, or sixth position of the glycoside ring), about the length of the oligolactide chains, and possibly about the total number of LA monomer units attached to the CD [15,18].

The ^1^H NMR spectrum presented in Figure S1 supports the structural assignment of the CD modified with LA, as depicted in Scheme 1. According to the results published by Shen et al., the esterification site is located on the β-CD smaller rim at the C6. The CD-LA derivatives with a higher substitution degree prepared in our work have, however, a different substitution pattern. Thus, we observed that some of the OH groups that remained unreacted were in positions 2, 3, and 6. The corresponding signals were flattened due to the esterification of the OH groups belonging to the neighboring glycoside units. The selectivity of the substitution at the OH group from position 6 would have determined the disappearance of the OH 6 signal, while the OH groups from positions 2 and 3 would have been still present. However, since the signals of the OH groups that remained unreacted in positions 2, 3, and 6 were noticed, we may consider that the CD was rather randomly esterified.

The number of lactate units per CD molecule may be theoretically calculated from the ratio between the resonance signal integrations corresponding with the CD and lactate. However, these calculations were prevented due to the resonance peak broadening of signals belonging to the modified CD and the overlapping between the peaks corresponding to various components (CD positional isomers derived from substitutions of CD at different positions—at C from positions 2, 3, or 6—of the modified CD mixture.

The ^1^H NMR spectra were, however, used for the calculation of the average length of the oligolactide chains attached to the CD. It was calculated from the ratio of the integral values belonging to the CH_3_ signals (*a’* at 1.49–1.41 ppm—in-chain methyl groups and *a* at 1.3–1.28 ppm—chain-end methyl groups). The (*a’ + a)/a* ratio was approximately 3/1, which signifies that the average chain length is 3 lactate units.

The CD substitution degree may be inferred from the GPC measurements, giving a relative average number molar mass of about 2400 g/mol (Figure S2). These values should be taken into consideration with the caveat that the *M*_n_ value determined by GPC may be significantly biased. The average number of lactate units per CD molecule may be precisely calculated by MALDI MS, as is shown later.

### 2.2. Mass Spectrometry (MS) Characterization

A deeper view into the structural features of the CD-LA product may be obtained by using mass spectrometry characterization. Therefore, we used two different MS methods: ESI Q-TOF MS and MALDI MS. A proper quantification of the *M*_n_ and *M*_w_ averages by ESI MS is prevented due to the formation of both single- and double-charged CD-LA corresponding peaks (Figure S3). On the other hand, the MALDI MS technique allows the observation of only single-charged species, thus allowing the calculation of the *M*_n_ and *M*_w_ averages.

**Figure 1:**
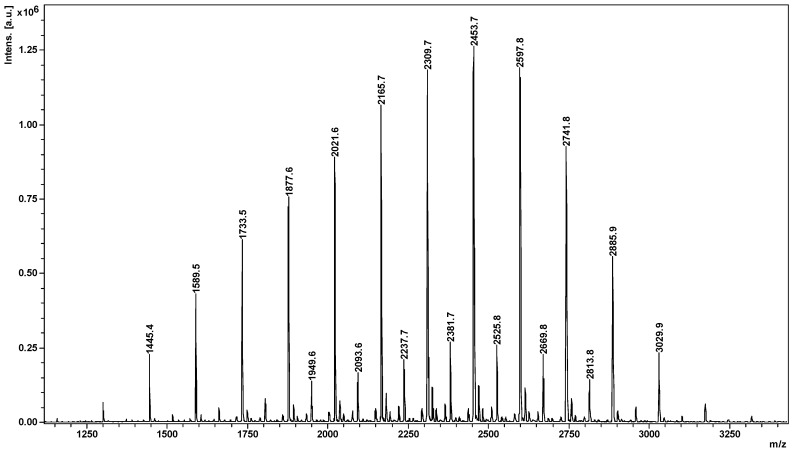
MALDI MS spectrum of CD-LA.

From the MALDI MS spectrum displayed in the Figure 1, we assigned the compounds corresponding to the spectrum peaks as CD-LA structures. The calculation of the CD-LA corresponding *m*/*z* values was performed using the formula *m*/*z =* 1134 (*CD*) *+ n ×* 72 (***la***) *+* 23 (*Na*). Thus, the base peak situated at *m*/*z* = 2453 was assigned to a CD derivative bearing 18 lactate units. There may actually be observed two series of peaks. The main series had a sequence of 144 Da (from lactide monomer unit), while the second series was shifted with 72 Da and may be described as having an odd number of lactate units. Therefore, these peaks corresponded to CD-LA chains issued from the transesterification processes leading to the formation of both types of chains containing odd and even numbers of lactate units. By comparing the intensity ratios of the peaks corresponding to the abovementioned series, we could observe that only a small proportion of the CD-LA products were undergoing transesterification reactions and the employed synthesis method had a low propensity for transesterification reactions. By taking into account the relative intensities of all the peaks of the spectrum of CD-LA, it was possible to calculate an average *M*_n_ value of 2280 g/mol, which corresponds to an approximate number of 7.8 of lactide or 15.6 lactate units per CD molecule, a bit less than the value obtained by GPC but in good agreement with the theoretical number of lactate units based on LA/CD feed ratio. 

Taking into account the NMR results, which gave the average length of the oligolactide chains attached to the CD (3 lactate units), and MALDI results, which gave the total number of lactate monomer units attached to the CD (15.6 lactate units), it was shown that CD-LA product had a star-like structure with an average of 5.2 arms with an average length of 3 lactate units each. Thus, the oligolactides were attached as multiple short chains to different OH groups of the CD molecule.

### 2.3. Fragmentation Studies by Collision-Induced Dissociation (CID) MS/MS on Electrospray (ESI) Q-TOF Instrument

In order to take the structural analysis to a deeper level, MS fragmentation studies (MS/MS or tandem MS) were performed. The aim of the tandem MS experiments was to establish a relationship between the observed fragmentation patterns and specific structural features of the analyzed compounds. These patterns would be further useful for structural identification at the molecular level of similar compounds.

Most of the MS fragmentation studies make use of ion trap and Q-TOF instruments which offer the possibility of precise isolation of the precursor ions. However, such detectors are usually associated with electrospray ionization due to construction- or application-related considerations. The drawback for polymer analysis is that sometimes the ion species targeted for fragmentation may not be obtained as single-charged species by ESI because of the propensity of the species with high molecular weight to form multiple charged species. Taking into consideration the abovementioned problems, we chose to analyze first the fragmentation profiles of a low molecular weight CD-LA product (monoisotopic precursor ions) by fragmentation in a Q-TOF instrument (collision-induced dissociation—CID) and then to check the fragmentation of higher molecular weight compounds by MALDI MS/MS via laser-induced dissociation (LID).

**Scheme 2:**
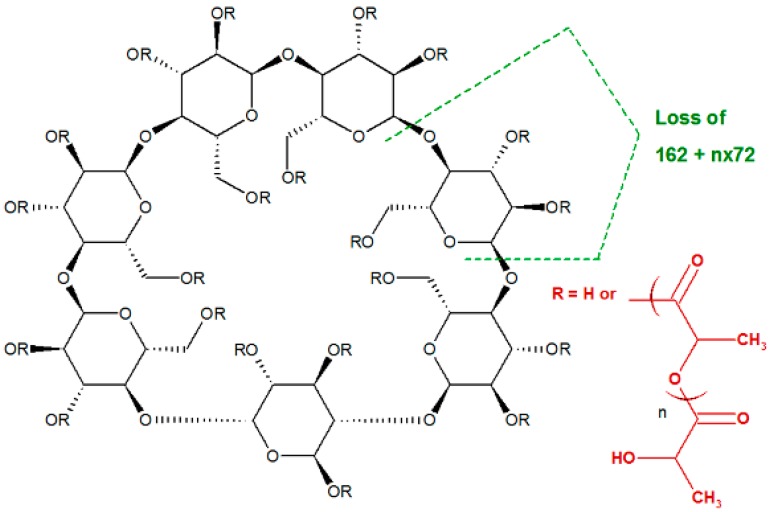
Fragmentation of the glycoside bonds with loss of the rings modified with variable number of lactate units—**G pathway** (R stands for H or lactate moieties).

The fragmentation of cyclodextrins and cyclodextrin derivatives through the cleavage of the semiacetalic bonds (Scheme 2), which results in daughter ions with a specific mass related to the number of glycoside structural units, remained in the composition of the respective fragments (m = n × 162 Da, where 162 Da represents the molecular mass of one glycoside unit and n is the number of structural units) [30].

**Scheme 3:**
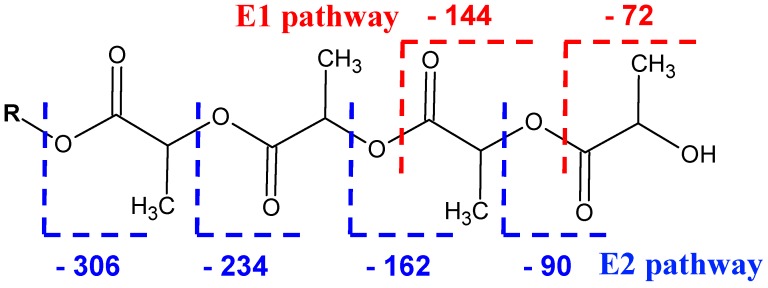
Fragmentation of the ester bonds at the level of the oligolactides chains attached to the CD–**E pathway**.

**Figure 2:**
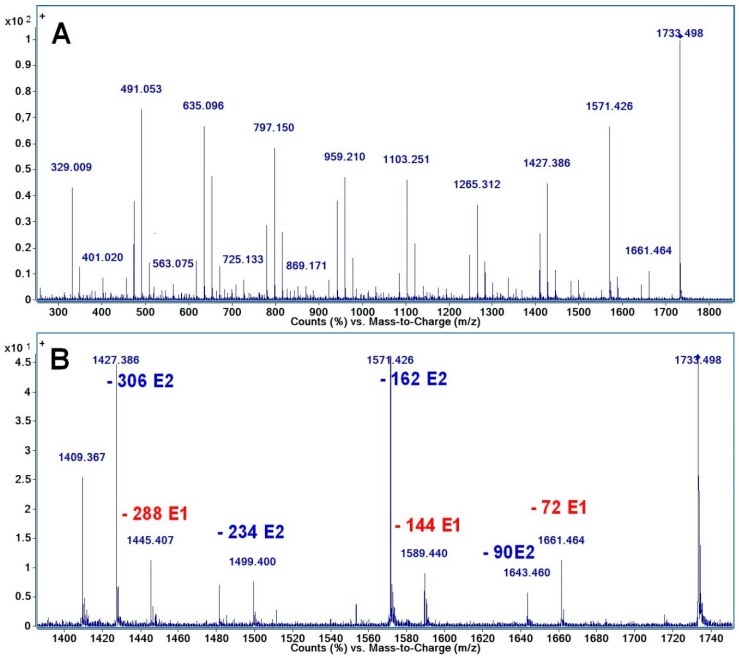
CID-MS/MS spectrum of [CD-LA_4_ + Na]^+^ precursor ions: (**A**)—full spectrum, (**B**)—enlarged region between *m*/*z*—1400–1740.

The fragmentation spectrum from Figure 2A of the CD-LA sodium-charged precursor ions having 8 lactate (4 LA units) units attached to the CD, noted as [CD-LA_4_ + Na]^+^ and having an *m/z* of 1733, consists of a series of peaks issued mainly from two types of cleavages: one at the level of semiacetalic bonds connecting the glycoside units (G pathway) (Scheme 2) and the second at the level of ester bonds on the alkyl and/or acyl side (E pathway) (Scheme 3).

The fragment peaks issued from the **G pathway** may be described as being composed of a variable number of attached glycoside units (n × 162 Da) and lactate units (m × 72). For example, the peak found at *m*/*z* = 959 may be assigned as a fragment containing 4 glycoside units and 4 lactate units, and thus may be denoted as [G_4_la_4_ + Na]^+^ and the *m/z =* 4 × 162 (***G***) *+* 4 × 72 (***la***) *+* 23. By analyzing the MS/MS spectrum from Figure 2A, it may be observed that the most intensive peaks correspond to the fragments containing even numbers of lactate units. The *m/z* values corresponding to these peaks are highlighted in the Table 1.

The second main fragmentation pathway underwent the cleavage of the ester bonds between the lactate units or the ester bonds connecting to the CD. Such fragmentations occurred at the acyl side (**E1**) and at the alchil side (**E2**) of the ester bonds [28], as shown in Scheme 3. The values of the neutral losses from both **E1** and **E2** series are annotated on the enlarged MS/MS spectrum in Figure 2B. Thus, the **E1** fragmentations proceeded by neutral losses of chains containing one or more lactate units (multiple of 72 Da). The **E2** fragmentations produced fragments with higher relative intensity by losses of chains ending in lactic acid and containing a variable number of lactate units [n × 72 (**la**) + 18 (H_2_O)].

Figure 2B shows that the mass of the fragment belonging to the **E2** series, produced by the loss of 162 Da (2 × 72 + 18), has the same mass as the fragment produced by the cleavage of one glycoside ring (**G** series) with the same neutral loss of 162 Da. The molecular formula of both the **G** and **E1** neutral lost fragments is C_6_H_10_O_5_ and they cannot be differentiated.

There was also a fragment peak situated at *m*/*z* = 1409 which may be formed by two simultaneous losses of 162 Da either by the **G** pathway alone (2 glycoside units), either by mixed fragmentations via **G** and **E2** pathways, or solely via **E2** by two concomitant losses of oligolactide chains each having a mass of 162 Da. The simultaneous loss of oligoester moieties attached to the CD was also observed in the case of CD modified with 3-OH butyrates [25]. In that case, the alternative fragmentation products were not isobaric peaks and therefore they were clearly observed.

### 2.4. Fragmentation by MALDI Laser-Induced Dissociation (LID)

**Figure 3:**
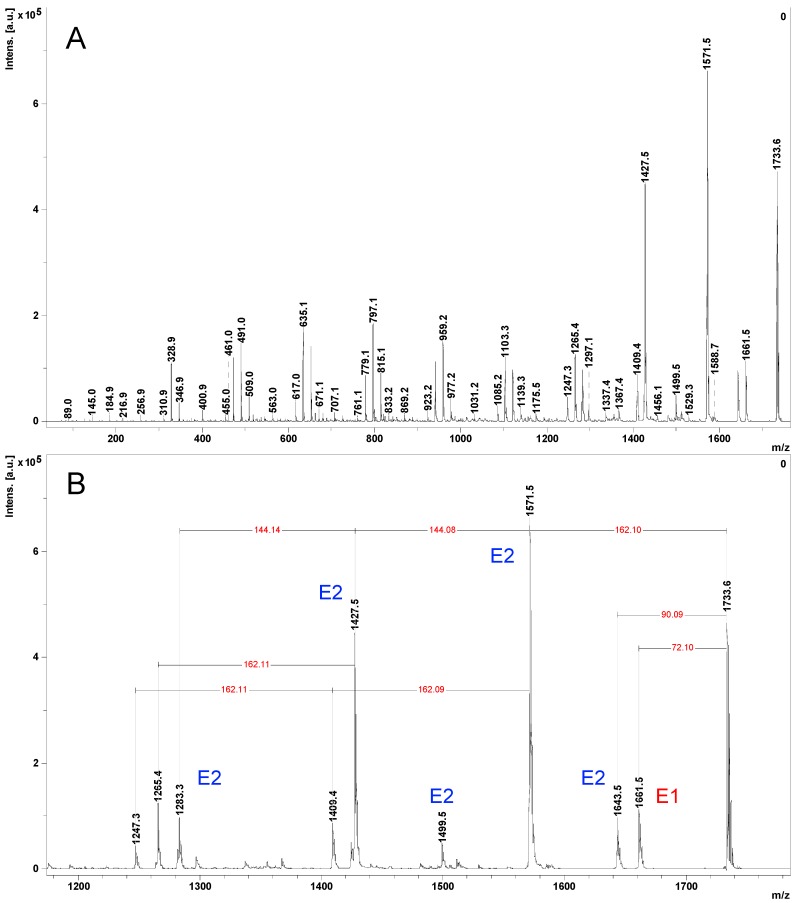
MALDI LID fragmentation spectrum of the [CD-LA_4_ + Na]^+^ precursor ions: (**A**)—full spectrum, (**B**)—enlarged region between *m*/*z*—1200–1800.

Next, we analyzed the MALDI MS/MS fragmentation of the same peak situated at 1733 Da—[CD-LA_4_ + Na]^+^ parent ions (Figure 3A). The comparison with Figure 2, presenting the CID MS/MS, obtained by using the ESI Q-TOF instrument, revealed that MALDI LID yields qualitatively similar fragments belonging to the **G** and **E2** series, while the **E1** series of peaks has a low relative intensity. The reduced complexity of the MALDI LID fragmentation profiles as compared with MALDI TOF/TOF CID profiles was also observed during the analysis of peptide fragmentations [31]. However, the relative intensity of the peaks belonging to the **G** series is obviously lower than the intensities of the fragment peaks belonging to the **E2** series probably because of the different fragmentation energetic conditions. Also, Figure 3B shows fragment peaks issued from two (*m*/*z* 1409 peak) or three (*m*/*z* 1247 peak) 162 Da simultaneous neutral losses (the 162 Da neutral losses are annotated). The presence of these peaks may originate, as explained earlier, from different simultaneous fragmentation processes. Such fragmentation comes to support the attachment of the oligolactides to CDs as multiple arms and not as a single chain, similar to what was observed after the analysis of ^1^H NMR spectra.

**Figure 4:**
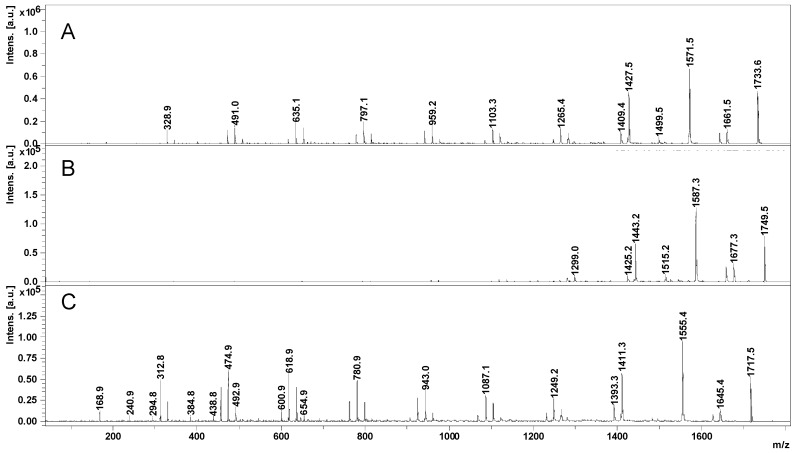
MALDI LID MS/MS of CD-LA_4_ precursor ions: Na (**A**), K (**B**), and Li (**C**) charged.

The fragmentation profiles of CD derivatives were noticed to slightly change according to the type of the cationization agent [25,32]. Thus, the fragmentation of the proton-charged triacetyl-β-CD yielded fragments from both cleavages of glycoside rings and ester bonds (**G** and **E pathways**) [25]. However, when sodium-charged triacetyl-β-CD ion species were subjected to CID MS/MS, there were observed mostly fragments issued from the cleavage of the ester bonds (**E pathways**). Therefore, we decided to compare the fragmentation profiles of Na- (*m*/*z* = 1733—M + 23), Li- (*m*/*z* = 1717—M + 7), and K- (*m*/*z* = 1749—M + 39) charged CD-LA_4_ parent ions (Figure 4).

**Figure 5:**
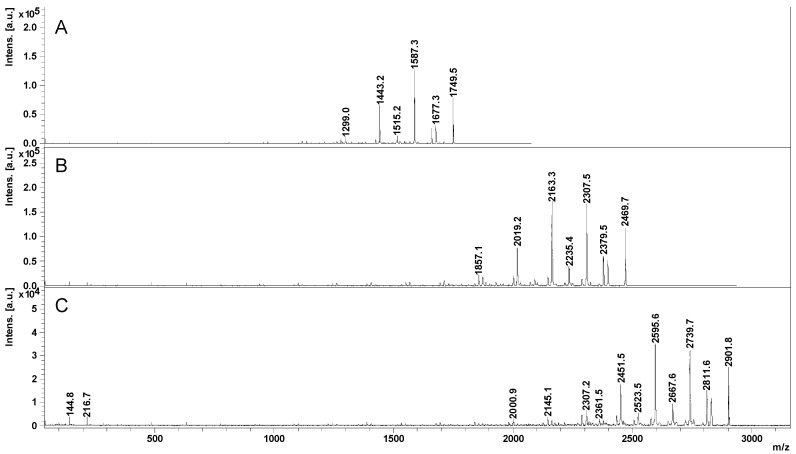
Comparative view of the MS/MS spectra of the CD-LA, K-charged parent ions: [CD-LA_4_ + K]^+^ (**A**), [CD-LA_9_ + K]^+^ (**B**), and [CD-LA_12_ + K]^+^ (**C**).

By analyzing the MS/MS spectra from Figure 4, it may be observed that the fragments resulting from the cleavage of the semiacetalic bonds (**G** series) were clearly appearing in the fragmentation profiles of Na^+^- and Li^+^-charged species, while the K^+^-charged parent ions yielded mostly fragments issued from the cleavage of the ester bonds (**E** series). The observed selectivity may be useful in analyzing the moieties connected via ester bonds to the CD. The propensity for ester fragmentations has been confirmed also for CD-LA with 18 lactate units [CD-LA_9_ + K]^+^ and CD-LA with 24 lactate units [CD-LA_12_ + K]^+^, as shown in Figure 5.

A particular feature of the observed fragmentation profile is the correlation of the number and relative intensities of the **E2** fragments with the previous structural information obtained by NMR and MS. First of all, the fragment peaks resulting from the loss of an odd number of lactate units have a lower relative intensity as compared with the series of fragments formed by the loss of an even number of lactate units. The odd-numbered lactate units in CD-LA solely appear via transesterification during the ROP process of LA**.** This fact supports the lower propensity of transesterifications for the ROP reaction system leading to the formation of the CD-LA product.

Also, the ^1^H NMR analysis revealed that the average length of the oligolactide chains in the CD-LA products is around 3 lactate units. It may be interesting to observe whether there is a correlation between the relative intensities of the **E2** fragments and the average length of oligolactide arms attached to the CD. Thus, considering that the **E2** fragments appear only from the cleavage of unique PLA chains (Scheme 3) and their relative intensity reflects their relative amount, we determined the average number of lactate units in a single chain attached to the CD. This calculation considered only the observed members of the **E2** series and was made with the caveat that specific energetic features of the MALDI LID fragmentation process may also influence the observed relative intensities of the considered fragment ions.

Thus, during the fragmentation of the CD-LA parent ions (Figure 5) with an increasing number of lactate units attached to the CD (from 8 to 24), there is an increasing number of lactate units which are neutrally lost. This may be the effect of the increase in the length of the PLA chains attached to the CD. The average number of lactate units obtained for the different fragmented parent ions is 2.6 lactate units for [CD-LA_4_ + K]^+^, 3.2 lactate units for [CD-LA_9_ + K]^+^, and 3.5 lactate units for [CD-LA_12_ + K]^+^. The calculations were based on the relative intensities of the fragment ions belonging to the **E2** series observed in the MS/MS spectra from Figure 5 (the detailed data for the peaks intensities taken into consideration are given in the Table S1). The average number of lost lactate units, *N_la_*, was calculated using the formula *N_la_* = Σ*n_i_I_ri_*/Σ*I_ri_*, where *n_i_* represents the number of lost lactate units and *I_ri_* represents the relative intensity of the considered fragment peak. Therefore, there may be inferred an average value of the PLA chain length of 3 lactate units, close to that obtained by ^1^H NMR. Thus, the MALDI MS and LID fragmentation experiments may constitute an alternative to the classical characterization techniques such as NMR and GPC for the structural confirmation of the CD-oligolactide derivatives. Further studies will aim to elucidate the conditions leading to such energetic selectivity for the differentiation of the fragmentation pathways in the CD-oligoester derivatives.

## 3. Materials and Methods

### 3.1. Chemicals

l-lactide (l-LA—Sigma-Aldrich, St. Louis, MO, USA) was recrystallized twice from ethyl acetate, dried under vacuum, and sublimated before use. β-cyclodextrin (CD—Cyclolab, Budapest, Hungary) was dried over P_2_O_5_ under vacuum at 80 °C for 72 h and kept over P_2_O_5_ in the desiccator under Ar atmosphere. The reaction solvent, dry dimethylformamide, was purchased from Aldrich and used as received. All other used solvents were HPLC grade and were used as received.

### 3.2. Solution Polymerization of L-LA in DMF

The CD-LA sample was prepared according to a previously reported procedure [19]. In a typical reaction, 2 g of CD and 2 g of L-LA (CD/L-LA 1:8 molar ratio) were weighted together and added into a flame-dried flask containing a magnetic stir bar and 30 mL of dry DMF, under protection of glove box. The flask, isolated with a rubber septum, was then taken outside the glove box and completely immersed in an oil bath over a heater with magnetic stirring and the temperature was brought to 85 °C. The L-LA monomer and the CD were completely dissolved in the reaction mixture after 1 h. The heating was maintained for 48 h under continuous stirring. The reaction was stopped by simply removing the flask from the heating source and leaving it to cool down for 30 min prior to purification. The samples were purified by repeated precipitation in cold diethylether and vacuum drying at 50 °C for 12 h to result in a fine white powder, yield 85%.

^1^H NMR (400.13 MHz, DMSO-d_6_, δ ppm): 5.94–5.70 (OH^2^, OH^3^), 5.5–5.42 (end chain OH), 5.20–5.12 (CH, in chain), 4.85 (H^1^), 4.65–4.18 (OH^6^, H^6’^, CH—end chain), 3.90 (H^5′^), 3.64–3.37 (H^3^, H^5^, H^6^, H^2^, H^4^), 1.49–1.41 (CH_3_, in chain), 1.3–1.28 (CH_3_, end chain).

MALDI MS: *M*_n_ = 2280 g/mol, *Đ* = 1.039

GPC: *M*_n_ = 2500 g/mol, *Đ* = 1.08

### 3.3. Matrix-Assisted Laser Desorption Ionization (MALDI MS)

The raw samples withdrawn directly from the polymerization mixture were dissolved in a 1/1 water/acetonitrile mixture to a concentration of 10 mg/mL. The liquid chromatography fractions were used as such. Samples were mixed with a matrix solution (saturated solution of α-cyano-hydroxy-cynamic acid in water/acetonitrile mixture) in a ratio of 1/100 (*v*/*v*). One microliter of this mixture was deposited on a polished steel MALDI target (Bruker, Bremen, Germany). Mass spectra of polymers were measured on an UltrafleXtreme TOF instrument (Bruker) equipped with a 355-nm smartbeam-2 laser capable of a pulsing frequency of 1 kHz. The mass spectrometer was operated by FlexControl 3.3 software (Bruker). Acquired spectra were processed by FlexAnalysis 3.3 software (Bruker). The ionization laser power was adjusted just above the threshold in order to produce charged species. The mass spectra were collected in an amount above 10,000 spectra for each sample. The fragmentation spectra were obtained in LIFT mode and 20,000 spectra were collected for each MS/MS spectrum. The samples were spiked with the desired cationization agent as 0.1 M solution in water (LiI, NaI, and KCl).

### 3.4. Electrospray Ionization Mass Spectrometry (ESI MS)

ESI MS experiments were conducted using the AGILENT 6520 LC QTOF MS equipped with a dual ESI source. The data were analyzed using the Mass Hunter software. The ESI MS parameters were set as follows: Vcap = 4000 V, fragmentor voltage = 200 V, drying gas temperature = 325 °C, drying gas flow = 10 L/min, and nebulizer pressure = 35 psig. Nitrogen was used as spraying gas. The samples were injected using a water/acetonitrile (1/1) solvent mixture at a 26 °C constant temperature in a column compartment. The used eluents were: A—2 mM formic acid solution and B—acetonitrile. The samples were solved in a 1:1 (*v*/*v*) water/acetonitrile mixture and 60 μL was injected.

CID MS/MS experiments were conducted using the AGILENT 6520 LC ESI QTOF mass spectrometer equipped with a dual ESI source. The fragmentation was performed using nitrogen as collision gas at a pressure of 18 psig inside the collision cell. The [CD-LA_4_ + Na]^+^ parent ions yielded fragment ions at variable E_lab_ = 120 eV. The sample was infused via an external syringe pump (KDS Scientific) with a flow of 0.05 mL/min. For the sodium charged parent ions, the samples were spiked with a 0.1 M NaI water solution.

### 3.5. Nuclear Magnetic Resonance (NMR)

The NMR spectra were recorded on a Bruker Avance DRX 400 MHz Spectrometer equipped with a 5-mm QNP direct detection probe and z-gradients. Spectra were recorded in DMSO-d_6_ at room temperature. The chemical shifts are reported as δ values (ppm) relative to the solvent residual peak.

### 3.6. Gel Permeation Chromatography (GPC)

The molecular parameters of CD-oligolactide covalent conjugates were also determined using a Shimadzu LC-20 isocratic pump and a Shimadzu refractive index detector in size exclusion mode using a PSS PFG precolumn and three PPS PFG columns (d = 8 mm, l = 300 mm) filled with particles of 7-μm size with pore sizes of 100, 300, and 1000 Å, respectively. Dimethylacetamide was used as an eluent. Poly(methyl methacrylate) standards were used for internal calibration.

## 4. Conclusions

The employed synthetic procedure allowed the synthesis of cyclodextrins modified with oligolactides—CD-LA—which were further used for detailed structural characterization in order to obtain information about the polymerization process from the point of view of CD acting as both catalyst and initiator as well as to develop a general analytical procedure applicable for detailed structural characterization of CD-oligoester derivatives. The synthesized CD-LA products were characterized by NMR, GPC, and advanced mass spectrometry techniques. The NMR supported the structural identification of CD-LA products as randomly esterified cyclodextrin with lactide oligomers having an average of 3 lactate units per chain. The MALDI MS analysis revealed the CD was substituted with an average of 15.6 lactate units per CD molecule corresponding, on average, to 5.2 oligolactide arms per CD molecule. Deeper insights into the structural architecture of CD-LA derivatives were provided using CID MS/MS by means of an ESI Q-TOF instrument. Thus, the structure of the CD-LA was accurately reflected in the observed fragmentation patterns. It was established that CD-LA derivatives undergo cleavages of bonds at the level of the oligosaccharide structure and also at the level of the PLA structure. Similar fragmentation patterns were observed during the fragmentation analysis using MALDI laser-induced fragmentation. The MALDI LID technique allowed the study of the MS/MS processes at higher molecular weights. Moreover, the CD-LA fragmentation profiles were shown to be influenced by the type of cationization agent employed—Li, Na, or K. Thus, the K-charged parent ions underwent cleavages selectively at the level of ester bonds, allowing us to ascertain the average length of the PLA chains attached to the CD. Overall, the employed characterization techniques allowed for the precise characterization of the structure of CD-oligomer covalent conjugates, situated at the border of low and high molecular weight compounds. The analytical procedure presented here can be beneficial for the accurate assessment of CD-oligoester structure. Such analysis may provide valuable information for correlating the structure with the properties of the materials based on the CD-oligoesters, such as inclusion complexes or even the electrospun mats, which may be further used for various applications, including biomedical, pharmaceutical, agricultural, etc.

## Figures and Tables

**Table 1:** The *m*/*z* values of the fragments issued from the semiacetalic bond cleavages; the values corresponding to the most intensive peaks are highlighted and the values of the peaks which were not found or were of low intensity are not highlighted.

G */la **	G_1_	G_2_	G_3_	G_4_	G_5_	G_6_	G_7_
**la_0_**	185	347	509	671	833	995	
**la_1_**	257	419	581	743	905	1067	
**la_2_**	329	491	653	815	977	1139	
**la_3_**	401	563	725	887	1049	1211	
**la_4_**	473	635	797	959	1121	1283	
**la_5_**	545	707	869	1031	1193	1355	
**la_6_**	617	779	941	1103	1265	1427	
**la_7_**	689	851	1013	1175	1337	1499	
**la_8_**	761	923	1085	1247	1409	1571	1733

* **G**—stands for the glycoside unit with the molecular mass of 162 Da and the subscript number describes the number of **G** units in the respective fragment. ** **la**—stands for the lactate unit with the molecular mass of 72 Da and the subscript number describes the number of **la** units in the respective fragment.

## Supplementary Materials

The following are available online at, Figure S1: 1H NMR spectrum of CD-LA, Figure S2: GPC results for the CD-LA, Figure S3: ESI MS spectrum of CD-LA (low molecular weight fraction), and Table S1: Fragment peak intensities (I_r_) observed in the MS/MS spectra of the K-charged precursor ions.
