# Supplementary material for: Structural Architectural Features of Cyclodextrin Oligoesters Revealed by Fragmentation Mass Spectrometry Analysis
Source: Molecules. 2018 Sep 5;23(9):2259. doi: 10.3390/molecules23092259 (PMC6225376; doi:10.3390/molecules23092259)
Supplement: Supplementary file 1 [file molecules-23-02259-s001.pdf]

Supporting Information

Structural Architectural Features of Cyclodextrin Oligoesters Revealed by Fragmentation Mass Spectrometry Analysis

Cristian Peptu [1,2,*], Maksym Danchenko [3], Ľudovít Škultéty [3] and Jaroslav Mosnáček [1,*]

[1] Polymer Institute, Slovak Academy of Sciences, Dúbravská cesta 9, 84541 Bratislava, Slovakia
[2] "Petru Poni" Institute of Macromolecular Chemistry, Grigore Ghica Voda 41A, 700487 Iasi, Romania
[3] Institute of Virology, Biomedical Research Center, Slovak Academy of Sciences, Dúbravská cesta 9, 84505 Bratislava, Slovakia; virumaks@savba.sk (M.D.); viruludo@savba.sk (Ľ.Š.)
* Correspondence: upolcris@savba.sk (C.P.); jaroslav.mosnacek@savba.sk (J.M.); Tel.: +421-2-3229-4353 (J.M.)

Received: 20 August 2018; Accepted: 3 September 2018; Published: 5 September 2018

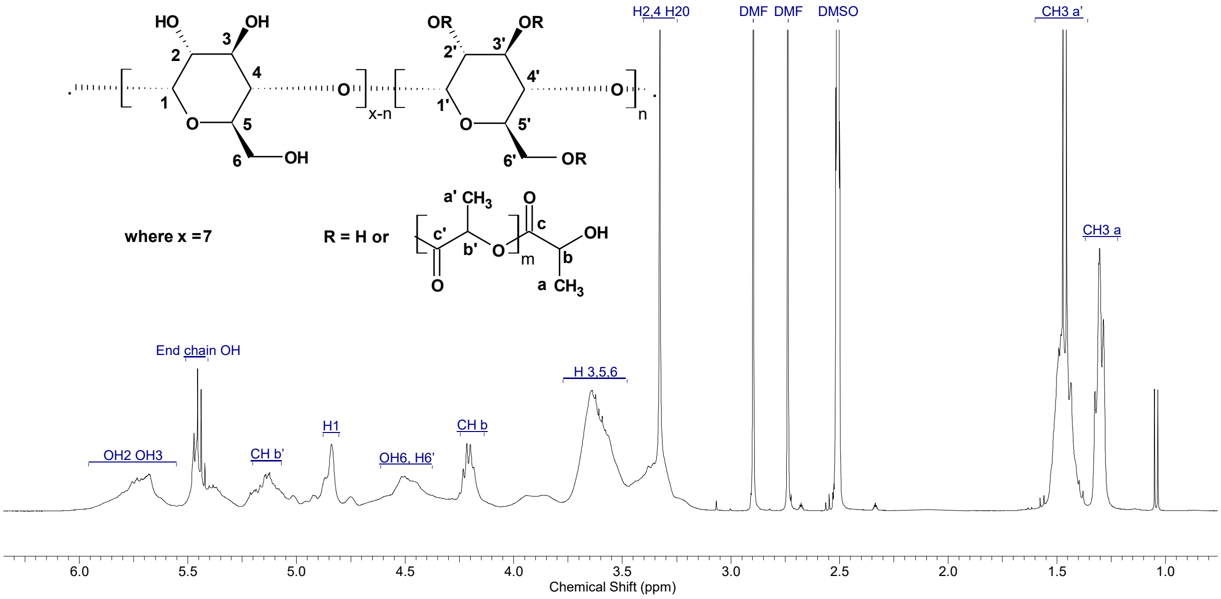

Figure S1. ^{1}H NMR spectrum of CD-LA.

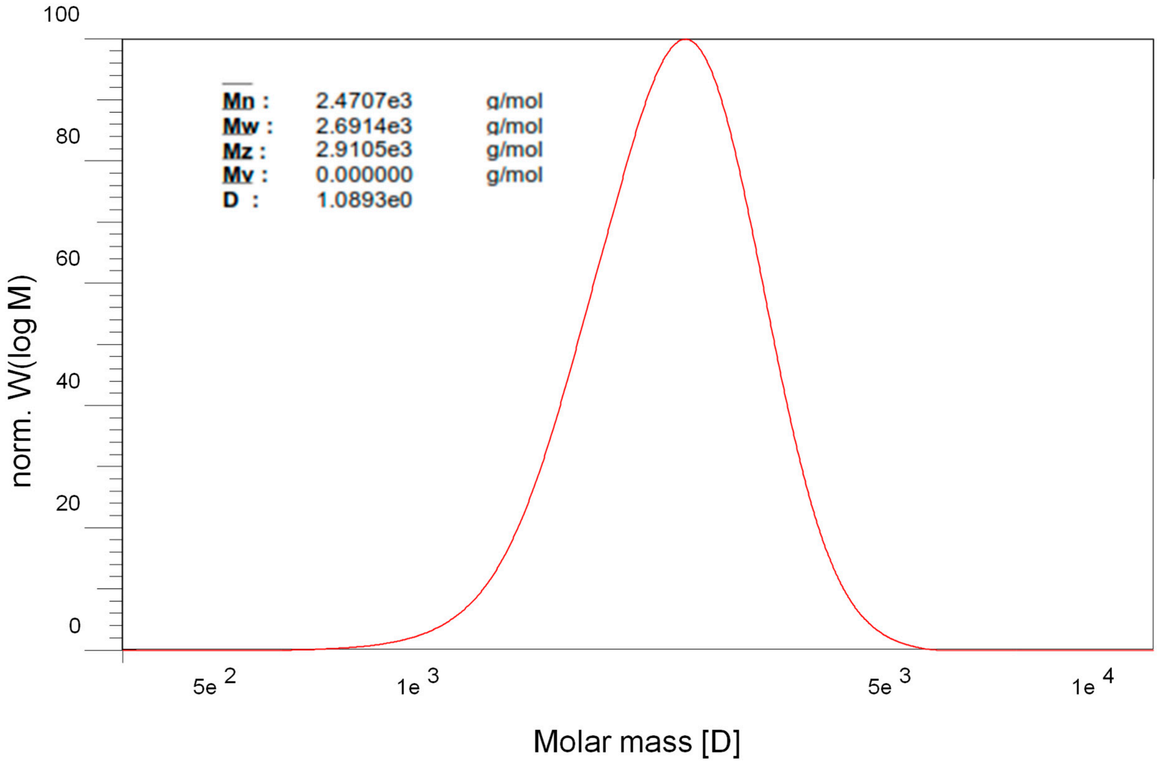

Figure S2. GPC results for the CD-LA.

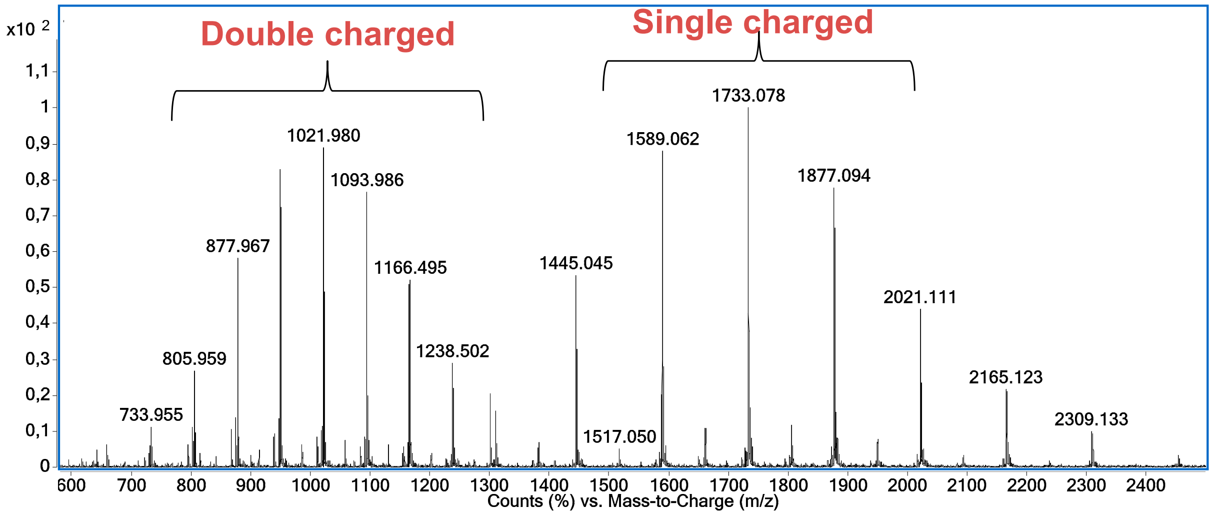

Figure S3. ESI MS spectrum of CD-LA (low molecular weight fraction).

Table S1. Fragment peak intensities (I_r) observed in the MS/MS spectra of the K-charged precursor ions.

Number of lost lactate units	I_r – [CD – LA_4 + K]$^+$	*m/z*	I_r – [CD – LA_9 + K]$^+$	*m/z*	I_r – [CD – LA_{12} + K]$^+$	*m/z*
**1**	23223	1659	47421	2379	9340	2811
**2**	106762	1587	134405	2307	20185	2739
**3**	9861	1515	29609	2235	6820	2667
**4**	55575	1443	141310	2163	24344	2595
**5**	-	-	-	-	3627	2523
**6**	7838	1299	64512	2019	13220	2451
